# Circadian, hormonal, and sleep rhythms: effects on cancer progression implications for treatment

**DOI:** 10.3389/fonc.2023.1269378

**Published:** 2023-09-07

**Authors:** Annemarie D. Jagielo, Catherine Benedict, David Spiegel

**Affiliations:** ^1^ PGSP-Stanford Psy.D. Consortium, Palo Alto University, Palo Alto, CA, United States; ^2^ Department of Psychiatry and Behavioral Sciences, School of Medicine, Stanford University, CA, Stanford, CA, United States

**Keywords:** circadian rhythms, cancer, sleep disturbance, cortisol, chronotherapeutics

## Abstract

Circadian, hormonal, and sleep rhythm disruptions are commonly experienced concerns among cancer patients throughout the cancer care continuum. This review aims to summarize the existing literature on circadian, hormonal, and sleep rhythms in the oncological population, focusing on circadian disruption and physiological and psychological abnormalities, disease progression, and chronomodulated treatment approaches. The findings demonstrate that subjectively and objectively measured circadian rhythm disruption is associated with adverse mental health and disease outcomes in patients with cancer. Chronomodulated chemotherapy, light therapy, cognitive behavioral therapy for insomnia, and physical activity have shown evidence of effectiveness in improving sleep, and occasionally, disease outcomes.

## 1 Introduction

Sleep is essential for health and wellbeing. Human beings have an internal 24-hour biological clock fluctuating from periods of sleep to wakefulness (1). During sleep, vital processes occur, including cellular repair, endocrine regulation, and memory consolidation in the brain (1). Disruptions of this sleep pattern can lead to detrimental physical health outcomes, such as the increased risk for diabetes, heart disease, obesity (2), neurodegeneration (3), and overall early mortality (4), as well as mental health challenges including depression (2).

Over half of people with cancer experience sleep disturbances (5), including difficulty in initiating and maintaining sleep, excessive daytime sleepiness, sleep-wake cycle dysregulation, and problems with sleep efficiency and quality (5, 6). Sleep disturbance has been identified as both a consequence of and a potential risk factor for cancer (7). Adjusting to a cancer diagnosis and treatment is stressful and may impact sleep quality (8); sleep disturbance may also be caused by factors such as medications and treatment side effects (9). Sleep disturbance throughout the cancer continuum from diagnosis to survivorship is associated with increased depression (10), fatigue (10), cognitive challenges (11), diminished quality of life (1, 12), and shortened survival (13).

## 2 Method

An independent review of the literature was performed by the first author using PubMed and Google Scholar databases. Eligibility criteria included peer-reviewed articles published in English that examined circadian and sleep disruption in the oncological population. Case studies and opinion and commentary papers were excluded. Search dates ranged from the earliest available date to 2023. The search themes were broadly categorized and included cancer-related circadian rhythm observations and associations, disruption and disease progression, and treatment approaches. The search terms included “cancer” and “circadian rhythms,” “circadian disruption,” “chronotherapy,” “CBT-I, “sleep disturbance,” “sleep-wake disorders,” “cortisol disruption,” “circadian disruption,” “circadian disruption and psychological outcomes,” “wrist actimetry,” “diurnal cortisol slope,” “light therapy,” and “physical activity.”

## 3 Results

### 3.1 Physiological and psychological abnormalities related to circadian disruption

#### 3.1.1 Sleep disorders in the oncological population

Oncological patients often suffer insomnia, hypersomnolence, sleep-disordered breathing, and sleep movement disorders (7, 14). In a cross-sectional survey examining the prevalence of sleep disturbance in cancer patients, 44% of patients reported fatigue, 41% endorsed restless legs, 31% had insomnia, and 28% reported excessive sleepiness (15). The prevalence of insomnia is two to three times greater among cancer patients compared to the general population (16, 17). Spielman’s three-factor model is comprised of predisposing, precipitating, and perpetuating factors of insomnia (18). In the cancer population, predisposing factors include age, family or personal history, comorbid psychiatric symptoms, and female sex (14, 19). Precipitating factors include cancer-related symptoms, emotional distress, hospitalization, and treatment (e.g., chemotherapy, surgery) which may disrupt patients’ sleep schedules (14, 19). Perpetuating factors include disease and treatment-related symptoms, poor sleep hygiene, daytime napping, irregular sleep schedules, and sleep myths (14, 19). Head and neck cancers and radiation treatment pose risk factors for obstructive sleep apnea, the most common form of breathing-related sleep disorders (20). Intermittent hypoxia may contribute to tumor growth and metastasis (21, 22).

Sleep disturbance is also closely associated with cancer-related fatigue (CRF), a separate but interrelated condition characterized by a profound sense of exhaustion that can severely disrupt patients’ quality of life, mood, and functioning (14, 23). CRF is associated with reduced daytime activity, increased daytime napping, and greater nighttime arousal (14, 24). CRF is a pervasive condition affecting 80-90% of patients undergoing cancer therapy and persists for months or years following treatment completion in roughly 30% of patients (23).

#### 3.1.2 Sleep disruption and psychological outcomes

Cortisol is a catabolic glucocorticoid hormone with a crucial role in the stress response. During periods of physiological or psychological threat, cortisol levels rise, preparing the body for a “fight or flight” response by mobilizing glucose into the blood (25–27). Cortisol levels commonly follow a diurnal cycle by peaking in the morning, declining throughout the day, and reaching a nadir at nighttime (28–30). The increase at night provides adequate blood glucose levels during the prolonged period of fasting at night – ‘those who sleep, dine’ (31–33). Circadian rhythms reflect the ability of the stress response system to function properly (34). Extended exposure to stress impairs the normal circadian rhythm of the hypothalamic-pituitary-adrenal (HPA) axis, resulting in elevated cortisol secretion throughout the day, when it normally declines.

#### 3.1.3 Disrupted circadian cortisol and psychiatric disorders

Salivary cortisol has been used as an objective biomarker to assess the HPA axis and circadian rhythm disruption as it measures the biologically active, unbound cortisol and is easier to access than blood draws (31, 35). Diurnal cortisol slope variation may be a useful marker of circadian function due to the relationship between circadian rhythms and cortisol (36). Aberrant cortisol rhythms are associated with negative psychological outcomes in the cancer population. For example, metastatic breast cancer patients who reported greater depressive symptoms demonstrated suppressed immunity as measured by lower average induration size in a test of cell-mediated immune response - delayed-type hypersensitivity to intradermal administration of seven common antigens (37). In advanced-stage ovarian cancer, higher cortisol levels (area under the curve) are associated with elevations in depressive symptoms and proinflammatory cytokine interleukin-6 (IL-6) and greater evening cortisol levels (38). Elevation of a single evening cortisol measure is a good marker of flattened diurnal cortisol levels in women with breast cancer (36). Elevated nocturnal cortisol and diminished cortisol variability are associated with enhanced functional disability, vegetative depression, and fatigue among ovarian cancer patients (31). Following primary treatment for epithelial ovarian cancer, diurnal cortisol rhythms normalized and IL-6 decreased (39), suggesting inflammation markers may re-regulate following treatment.

Psychological distress and coping styles can impact circadian rest/activity and cortisol rhythms. Giese-Davis et al. (28) demonstrated that metastatic breast cancer patients who repressed emotion displayed flatter cortisol rhythms compared to self-assured and nonextreme groups. Similarly, distress and avoidant coping are associated with circadian rest/activity rhythm disruption in presurgical breast cancer patients (40), while avoidance-oriented coping is associated with flattened diurnal cortisol slopes in prostate cancer survivors (41). There is a significant relationship between posttraumatic growth and diurnal cortisol slope in metastatic breast cancer patients, suggesting positive psychological changes may be associated with normalization of cortisol slope and enhanced endocrine functioning (42). These results suggest the psychological sequelae associated with cancer may be related to the dysregulation of the HPA axis.

#### 3.1.4 Subjective sleep measures and psychological outcomes

Sleep disturbance negatively impacts quality of life in patients with mixed cancers (43), breast cancer (44), and ovarian cancer (45). Sleep disturbance is significantly correlated with self-reported depression and fatigue in mixed cancer patients (10), while depression, pain, and life stress predict sleep disturbance in metastatic breast cancer patients (46). Poor sleep quality leads to emotional dysregulation and impaired daily functioning in patients with breast cancer (44). Daytime fatigue and diminished quality of life are qualities associated with shorter cancer survival (47). For patients with lung cancer, frequent nighttime arousals are associated with higher mortality risk (13). In many studies, sleep disturbance occurred early in the illness course (10, 13, 45). Some might expect sleep disturbance to occur solely at the end of life when numerous systems are disrupted. However, these data suggest sleep disturbance may not be the result of having a terminal illness, but rather an earlier factor in the disease progression.

### 3.2 Circadian disruption and disease progression

#### 3.2.1 Circadian hormonal disruption and tumor growth

Circadian rhythm disruption may impact the ability of the immune system to fight tumor growth. A relationship exists between cancer and circadian clock genes, growth control, and growth effector genes (34). The circadian timing system (CTS) is a molecular clock with at least fifteen genes that regulate the sleep-wake cycle (48, 49). High-quality sleep reflects a robust CTS (47). CTS genes are critical for generating circadian rhythms, while disruptions in this system may promote tumor progression. Indeed, the mPer2 gene plays a crucial role in the circadian clock, and mPer2 gene deficiencies are related to tumor growth in mice (34, 50). Meal timing has been used to reset and reinforce the CTS (48, 51, 52). In mice, the use of meal timing enhances survival (51) and inhibits cancer growth (52). The clinical significance of these findings is supported by a study of 361 patients with colorectal cancer (53). Sleep problems at baseline independently predicted a higher risk of earlier death (HR: 1.36; p = 0.011), disease progression (HR: 1.43; p = 0.002) and poor treatment response (RR: 0.58; p = 0.016). These findings suggest circadian disruption may lead to cancer growth.

#### 3.2.2 Circadian hormonal disruption and cancer progression

The disruption of circadian HPA rhythms is associated with cancer progression (34, 54–59). Patients with advanced cancer often demonstrate flattened diurnal cortisol compared with healthy controls (54, 55, 60, 61). Flattened cortisol slope is a prognostic indicator of early mortality in patients with breast cancer (61), lung cancer (54), and ovarian cancer (62). Abnormal cortisol rhythms characterized by a less rapid decline in cortisol levels late in the day are associated with fatigue in breast cancer survivors as well (63).

#### 3.2.3 Sleep disruption and cancer progression

The actigraphic dichotomy index I<O is a robust metric that reflects individuals’ daily patterns by indicating the percentage of in-bed activity counts that are less than median of out-of-bed counts (48, 64–69). A greater I<O index reflects enhanced circadian function and reduced nighttime motor activity (65, 69). I<O is a risk factor for overall survival in metastatic colorectal cancer (48) and breast cancer (70). However, findings regarding the relationship between circadian disruption measured through wrist actimetry and patient-reported subjective sleep data appear to be mixed, although subjective reports of poor sleep are necessarily somewhat unreliable (65, 71–73). Many studies noted a discrepancy between subjective and objective measurements (65, 71, 72) or inconsistent findings (74); yet Grutsch et al. (47) found a correlation between rest/activity rhythms and patient self-reported sleep quality on the Pittsburgh Sleep Quality Index (PSQI) questionnaire. This represents an area for further investigation and highlights the importance of acquiring both objective and subjective patient sleep data.

### 3.3 Treatment

#### 3.3.1 Chronomodulated chemotherapy

Chronomodulated chemotherapy is the timed administration of chemotherapy based on circadian rhythms to enhance anticancer drug efficacy and/or tolerability and to reduce side effects (68, 75, 76). Modifying the timing of chemotherapy administration based on physiological rhythms can enhance treatment outcomes for patients with cancer (68, 76, 77). In a systematic review of 18 randomized controlled trials conducted by Printezi et al. (75), the use of chronomodulated chemotherapy was associated with reduced toxicity in 61% of studies. Furthermore, 17% of studies demonstrated enhanced efficacy of chronomodulated chemotherapy, as evidenced by overall survival, objective response rate, or time to treatment failure. However, in 11% of studies, chronomodulated chemotherapy reduced some toxic effects but increased others, and one (6%) study reported worse toxicity effects with chronomodulated chemotherapy compared to traditional chemotherapy. Thus, in most studies, chronomodulated chemotherapy reduced toxicity without necessarily enhancing efficacy (75). A meta-analysis of three phase III trials showed males but not females with metastatic colorectal cancer obtained a survival benefit with chronomodulation vs. conventional chemotherapy (78). Chronomodulated chemotherapy has not yet been widely adopted despite these findings (79).

#### 3.3.2 Light therapy

Light serves as a zeitgeber for the human clock (80). Light therapy can synchronize circadian rhythms and it is used to treat disorders linked to circadian disruption, such as shift-work syndrome, seasonal depression, fatigue, and jet lag (81–86). Light therapy may reduce circadian deterioration during chemotherapy and throughout survivorship. Studies show its benefits in preventing circadian rhythm decline (87, 88) and reducing CRF (81) during chemotherapy. A randomized controlled trial found bright light therapy led to an improvement in cancer survivors’ CRF compared to dim red-light exposure (89). A recent randomized controlled trial (83) evaluated the efficacy of bright light therapy compared to dim white light in a sample of 166 (non-)Hodgkin lymphoma survivors. Participants in both conditions reported reductions in CRF and improvements in mood, sleep quality, and quality of life. These results suggest light therapy may serve as a promising intervention to regulate circadian clocks.

#### 3.3.3 Cognitive behavioral therapy for insomnia

Insomnia treatment often includes a pharmacological component. Sedative hypnotics and antidepressants are frequently prescribed (14, 90), but their evidence base is lacking in the oncological population (7, 14). The use of these medications is not a recommended long-term strategy (14). If incorporated into treatment, pharmacotherapy is recommended in combination with Cognitive Behavioral Therapy for Insomnia (CBT-I; 14). CBT-I is the first-line treatment for insomnia, compromising relaxation training, sleep hygiene, cognitive restructuring, stimulus control, and sleep consolidation (91). CBT-I can be delivered in-person, via Telehealth, or self-administered by video, improving access to care (92, 93). CBT-I has demonstrated efficacy in the cancer population. For example, a meta-analysis of 16 trials found CBT-I improved various insomnia outcomes, including insomnia severity, sleep late onset, wake after sleep, sleep time, and sleep efficiency (94). However, these effects were temporary and diminished in short-term follow-up. Similarly, a meta-analysis examining the efficacy of CBT-I for cancer survivors found CBT-I improved patients’ insomnia severity, sleep efficiency, sleep latency, and wake after sleep onset, with effects remaining durable for up to 6 months (95). A comprehensive review of 12 studies found CBT-I delivered in various modalities improved cancer patients’ insomnia outcomes, mood, quality of life, and CRF (92). CBT-I was more effective than mindfulness-based cancer recovery in changing patients’ dysfunctional beliefs regarding sleep (96), although both interventions reduced insomnia severity. Self-administered CBT-I is a cost-effective alternative to professionally based delivery, though slightly less effective (93). Taken together, these results suggest CBT-I has merit in improving cancer patients’ sleep outcomes throughout the cancer continuum.

#### 3.3.4 Physical activity

In the general population, physical activity has been shown to improve sleep (97, 98). Exercise may serve as a preferable, low-cost, and readily implementable lifestyle modification for individuals with sleep-related problems. Exercise has been shown to improve the subjective and objective sleep quality of cancer patients and survivors (99–102). Although research is limited in the cancer population, studies have demonstrated yoga (101), tai chi (102), light intensity (100), and wearable technology-based physical activity (99) can reduce sleep disturbance and improve sleep quality in the oncological population.

## 4 Discussion

### 4.1 Strengths and limitations

The literature on circadian disruption and psychological outcomes demonstrates a significant relationship between psychosocial factors and sleep disturbance in cancer patients. A strength of the literature is the use of biological indicators such as diurnal cortisol slope to assess circadian disruption (28, 29, 31, 36–42). Additionally, Schrepf et al. (39) and Hoyt et al. (41) utilized longitudinal designs, allowing for observations of changes in diurnal cortisol and outcomes over time. Limitations include the cross-sectional and correlational study designs, limiting causal inferences, and lack of inclusion of healthy control groups in most studies (28, 31, 36–42). Objective biomarkers (i.e., diurnal cortisol slope and wrist actimetry) bolster research on circadian disruption and cancer progression. Wearable biomarkers provide an affordable and accessible means for assessing circadian disruption (66, 71). However, the correlational nature of the literature restricts causal connections, while small sample sizes (54, 56, 59, 63, 70) and homogenous samples (62, 70) limit generalizability.

The chronomodulated chemotherapy literature is strengthened by the use of randomized controlled trials to demonstrate causal effects of toxicity reduction following treatment (75). Limitations include the relative paucity of available research on this subject, heterogeneous study designs, and varied dosage/duration of infusions in the literature that prevented Printezi et al. (75) from examining the independent impact of chronomodulation. The fact that chronomodulation seems to work better among male vs. female patients introduces a source of variability in response that is not yet fully understood (78). Strengths of the light therapy research include the RCT designs (81, 83, 87–89) and the use of both objective and subjective measures to assess sleep/wake patterns (81, 83, 88, 89). Limitations of the studies include relatively small sample sizes (81, 87, 88) and limited adherence to the lightbox intervention (81, 87).

Strengths of the CBT-I literature include the number of meta-analyses included in the systematic review of CBT-I by Gao et al. (94) and the inclusion of systematic searching and methods sections in the other systematic review articles (92, 95). Limitations include the significant number of patients who do not adhere to the CBT-I protocol (92) and the potential risk of publication bias (92, 94, 95). Finally, strengths of the physical activity literature include the use of actigraphy data to assess objective sleep/physical activity patterns (100, 101), the novelty of the wearable technology-based intervention (99), the use of objective and subjective measures both pre- and post-intervention (99, 101), and random assignment and a partially blinded treatment protocol (102). The limitations include homogenous (102) and relatively small samples (99, 100, 102), lack of a triple-blind study design (101), and cross-sectional study design (100).

### 4.2 Future directions for research and treatment

Circadian-based chemotherapy administration is an important therapeutic option that may reduce chemotherapy toxicity (75). However, larger randomized controlled trials are needed to inform clinical practice (75). Novel, machine-learning approaches to assess circadian clock disruption in oncology are currently being developed (103, 104) and may have promise as a prognostic biomarker in breast cancer. These circadian-conscious strategies may refine cancer treatment moving forward. Overall, these findings have significant clinical implications and suggest providers should assess for sleep disruption throughout the cancer trajectory and utilize relevant, evidence-based treatments when clinically indicated.

### 4.3 Conclusion

There is a clear association between circadian disruption and adverse psychological and medical consequences in the oncological population. This review provides evidence that diurnal cortisol variation is associated with many psychological abnormalities, such as depression and fatigue. Further, this review underscored associations between circadian disruption and disease progression. Flattened cortisol slope is a prognostic indicator in a variety of cancer types, while I<O abnormalities constitute a risk factor in metastatic colorectal and breast cancer. Treatments including chronomodulated chemotherapy, light therapy, CBT-I, and physical activity have demonstrated evidence of efficacy in improving sleep, reducing treatment toxicity, and, in some cases, disease outcomes.

## Author contributions

AJ: Writing – original draft, Writing – review & editing. CB: Writing – review & editing. DS: Writing – review & editing.
